# Mapping Alveolar Oxygen Partial Pressure in COPD Using Hyperpolarized Helium-3: The Multi-Ethnic Study of Atherosclerosis (MESA) COPD Study

**DOI:** 10.3390/tomography8050190

**Published:** 2022-09-13

**Authors:** Naz P. Taskiran, Grant T. Hiura, Xuzhe Zhang, R. Graham Barr, Stephen M. Dashnaw, Eric A. Hoffman, Daniel Malinsky, Elizabeth C. Oelsner, Martin R. Prince, Benjamin M. Smith, Yanping Sun, Yifei Sun, Jim M. Wild, Wei Shen, Emlyn W. Hughes

**Affiliations:** 1 Department of Chemical Engineering, Columbia University, New York, NY 10027, USA; 2 Division of General Medicine, Columbia University Irving Medial Center, New York, NY 10032, USA; 3 Department of Biomedical Engineering, Columbia University, New York, NY 10027, USA; 4 Neurological Institute, Radiology, Columbia University, New York, NY 10032, USA; 5 Department of Internal Medicine, University of Iowa, Iowa City, IA 52242, USA; 6 Mailman School of Public Health, Columbia University, New York, NY 10032, USA; 7 Department of Radiology, Weill Cornell Medicine, New York, NY 10065, USA; 8 Department of Medicine, McGill University, Montreal, QC H3G 2M1, Canada; 9 Department of Infection, Immunity and Cardiovascular Disease, University of Sheffield, Sheffield S10 2TN, UK; 10 Division of Pediatric Gastroenterology, Hepatology and Nutrition, Department of Pediatrics, Columbia University Irving Medical Center, New York, NY 10032, USA; 11 Institute of Human Nutrition, College of Physicians & Surgeons, Columbia University Irving Medical Center, New York, NY 10032, USA; 12 Columbia Magnetic Resonance Research Center (CMRRC), Columbia University, New York, NY 10027, USA; 13 Department of Physics, Columbia University, New York, NY 10027, USA

**Keywords:** quantitative MRI, COPD, average diffusion coefficient, partial pressure of oxygen, hyper polarized gas MRI

## Abstract

Chronic obstructive pulmonary disease (COPD) and emphysema are characterized by functional and structural damage which increases the spaces for gaseous diffusion and impairs oxygen exchange. Here we explore the potential for hyperpolarized (HP) ^3^He MRI to characterize lung structure and function in a large-scale population-based study. Participants (*n* = 54) from the Multi-Ethnic Study of Atherosclerosis (MESA) COPD Study, a nested case-control study of COPD among participants with 10+ packyears underwent HP ^3^He MRI measuring p_A_O_2_, apparent diffusion coefficient (ADC), and ventilation. HP MRI measures were compared to full-lung CT and pulmonary function testing. High ADC values (>0.4 cm^2^/s) correlated with emphysema and heterogeneity in p_A_O_2_ measurements. Strong correlations were found between the heterogeneity of global p_A_O_2_ as summarized by its standard deviation (SD) (*p* < 0.0002) and non-physiologic p_A_O_2_ values (*p* < 0.0001) with percent emphysema on CT. A regional study revealed a strong association between p_A_O_2_ SD and visual emphysema severity (*p* < 0.003) and an association with the paraseptal emphysema subtype (*p* < 0.04) after adjustment for demographics and smoking status. HP noble gas p_A_O_2_ heterogeneity and the fraction of non-physiological p_A_O_2_ results increase in mild to moderate COPD. Measurements of p_A_O_2_ are sensitive to regional emphysematous damage detected by CT and may be used to probe pulmonary emphysema subtypes. HP noble gas lung MRI provides non-invasive information about COPD severity and lung function without ionizing radiation.

## 1. Introduction

Chronic obstructive pulmonary disease (COPD) was the third-leading cause of death globally in 2019 [1]. COPD is typically identified by lung function tests such as spirometry where low values of the ratio of forced expiratory volume at one second to forced vital capacity (FEV_1_/FVC) provide a primary signature. Despite decades of COPD research, there has been surprisingly little progress towards reducing COPD mortality, indicating a need for better comprehension of disease progression and pathophysiology. Textbook symptomatic characterizations of the disease, such as “Blue Bloater” and “Pink Puffers”, were established six decades ago [2,3]. COPD characterizations beyond symptomatic measures, using more advanced technologies such as computed tomography (CT) and modern-day machine learning algorithms are rising in importance for the identification of emphysema subtypes [4,5]. However, CT requires radiation exposure and measures only anatomy, not pulmonary function.

Since the first magnetic resonance imaging (MRI) measurements using hyperpolarized (HP) noble gas a quarter of a century ago [6], MRI of human lung parenchyma has provided a new, safe, non-invasive way of exploring acinar structure and functional changes occurring in COPD [7,8,9,10]. HP MRI probes, among other parameters, ventilation defects, acinar structure characterizations via apparent diffusion coefficient (ADC) measurements, and direct determination of the regional alveolar partial pressure of oxygen (p_A_O_2_). Many pulmonary diseases and COPD, in particular, are characterized by ventilation-perfusion mismatch, where p_A_O_2_ and regional ventilation represent a vital input [11]. Over the past 20 years, improvements in HP ^3^He MRI have occurred from pilot studies on relatively small numbers of participants, beginning primarily with healthy smokers [12,13] and eventually including subjects with COPD [14,15,16]. Early studies established the first p_A_O_2_ maps of lung parenchyma for COPD participants [17]. However, since p_A_O_2_ measurements require observation of the decay of the HP ^3^He signal over a long breath-hold (10–20 s), technical challenges arise especially as the extent of damage increases. In particular, ^3^He gas flowing to neighboring voxels during the p_A_O_2_ acquisition results in partial pressure measurements that are negative [18]. Subsequent studies using HP ^3^He and more sophisticated multi-breath imaging techniques [19] partially alleviate this difficulty, but with some loss in the signal-to-noise ratio (SNR). In parallel to studies of p_A_O_2_ from HP ^3^He MRI, ADC measurements on COPD participants have reached a quite advanced stage of development and suffer less from systematic uncertainties and provide a metric complementary to CT data for characterizing emphysema subtypes. Gas travels with Brownian motion inside the lung due to thermal energy. While gas velocity remains constant between subjects, ADC reflects how far gas molecules travel during the data acquisition which is indicative of acinar microstructure. Modeling and parameter determination at the 10 mm to 100 mm scales reveal significant sensitivity to changes from COPD and other lung diseases where alveolar damage occurs [20,21,22,23,24,25,26]. Multiparametric clinical studies using ADC measurements to characterize COPD have recently become available [5,27,28,29].

There is a significant gap in knowledge pertaining to the study of COPD using hyperpolarized noble gas and particularly to p_A_O_2_. The MESA COPD studied an overall larger number of participants, particularly more with mild disease as compared to the more extreme cases in the current literature [4]. In this study, negative p_A_O_2_ measures were not discarded as non-physiologic but rather included in calculations and used as a metric to quantify the extent of disease. Finally, the emphysema subtypes classification is a novel approach aiming to deepen the understanding of different types of disease progression [4].

In this paper we explore the potential for HP ^3^He MRI in COPD in a large-scale population-based study comparing to CT, spirometry, diffusion capacity of carbon monoxide (DLCO) and other measures of pulmonary function. We use ADC measurements as a baseline metric for COPD characterization, where high ADC values reflect emphysematous damage increasing alveolar spaces to allow more room for gas motion. We investigate p_A_O_2_ measurements to quantify the impact of COPD on ventilation and gas exchange. In addition, we compare p_A_O_2_ results with emphysema subtypes assessed in six zones on CT [4].

## 2. Materials and Methods

### 2.1. Participants

The Multi-Ethnic Study of Atherosclerosis (MESA) COPD Study was a multicenter case-control study of COPD [30,31] nested in MESA, a population-based prospective cohort study of subclinical atherosclerosis, and the Emphysema and Cancer Action Project (EMCAP), a non-overlapping lung cancer screening study and community-based controls. Inclusion criteria were age 50 to 79 years and 10 or more pack-years of smoking; participants with contraindications to MRI and gadolinium were excluded. Known cardiovascular disease was excluded based on both the MESA and the MESA COPD protocols. All participants at one site were invited to undergo HP ^3^He MRI. Table 1 summarizes the characteristics of the study population. Institutional Review Board approval was obtained for all activities and all participants provided written informed consent.

### 2.2. HP ^3^He Production and MRI Hardware

Hyperpolarized ^3^He was produced via spin-exchange collisions with optically pumped polarized rubidium vapor in a glass tube at ~160 °C using a circularly polarized 100-watt diode laser emitting at 795 nm [32]. The ^3^He polarizer (GE Healthcare, Princeton, NJ, USA) produced ~1.3 L of polarized ^3^He gas, per batch, with polarization fractions ranging from 25% to 40% after 12 to 16 h (typically overnight) of optical pumping. The HP ^3^He gas was dispersed mixed together with ultrapure nitrogen, see Table 2, in 1 L batches using 1-L Tedlar bags.

^3^He imaging was performed on a Phillips Achieva 3 T multi-nuclear scanner tuned to the ^3^He central frequency at 97.32 MHz. Images were acquired using a flexible quadrature transmit-receive chest coil with passive proton decoupling for proton MRI. The chest coil dimensions were 35 cm length and 120 cm circumference matched to an adult torso. A thermal calibration source consisting of a half-liter bottle of ~3 bar of unpolarized ^3^He was used to set the central frequency.

### 2.3. MRI Acquisition

Each participant inhaled a 1 L mixture of HP ^3^He and ultrapure nitrogen for a 20-s breath-hold p_A_O_2_ scan starting at residual volume (RC) so imaging occurred at approximately functional residual volume (FRC). Some participants did not inhale exactly 1 L above RC and ended up breath holding for the scan at or slightly above FRC. The pulse sequence timing followed the work of Marshall et al. [18] with an initial time delay between the first two acquisitions, t_1_ = 1.3 s, and subsequent data collections following a time spacing of t_2_ = 4.5 s for a total of six acquisitions.

The second acquisition performed after τ_1_ was used to correct for RF depolarization [33,34]. The p_A_O_2_ imaging parameters were echo time TE = 0.48 ms, repetition time TR = 4 ms, field of view FOV = 350 mm, in-plane matrix 256 × 256, bandwidth = 127.5 kHz, slice thickness = 16 mm and flip angle = 1°. Twelve coronal slices were collected per subject.

Coronal proton MRI as well as HP ^3^He MRI ventilation scans were collected in the same 20 s breath-hold. Proton scans were used to define the lung boundaries and create ventilation masks as well as identify different regions within.

^3^He signal depolarizes over time primarily due to the presence of oxygen, such that magnetization, M_n_, follows an exponential decay given by [35]

M_n_ = M_0_cos^n·N^(α)·exp [−t_n_(k)/T_1_] (1)

where M_0_ and M_n_ are ^3^He magnetizations initially and after n slices, respectively. The flip angle is α, t_n_(k) is the time of acquisition n of the kth slice, and N is the number of phase-encoded steps. An exponential fit to the data provides a determination for T_1_ decay for each voxel. The decay time T_1_ is directly related to p_A_O_2_ via

p_A_O_2_ = ξ/T_1_ (2)

The constant ξ is experimentally determined in a controlled calibration measurement [17,36], which yields at body temperature (37 °C), ξ = 2.61 bar·s. 

For the ADC measurements, 350 mL of ^3^He was combined with 650 mL of ultrapure nitrogen and inhaled to total lung capacity (TLC). Coronal scans were taken at two different b values: b_0_ = 0 and b_1_ = 1.6 s/cm^2^. The relationship between ADC value and the signals, S_0_ and S_1_ from the two scans, is given by

(3) $$\mathrm{ADC}=\frac{1}{b_{1}}\log\frac{S_{0}}{S_{1}}$$

The scan parameters for the ADC measurements in this study were TE = 0.49 ms, TR = 120 ms, matrix size 128 × 128, FOV = 450 mm, slice thickness 40 mm, number of slices = 5, bandwidth = 63.7 kHz, and the flip angle = 3°. ADC measurements were performed at total lung capacity (TLC).

### 2.4. Co-Registration of HP ^3^He ADC with p_A_O_2_

**Figure 1:**
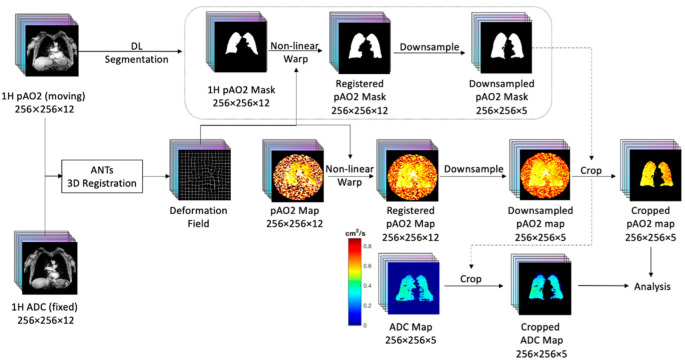
Workflow for the lung MRI co-registration between different breath stages.

Proton MRI scans acquired within the same breath hold for ADC and p_A_O_2_ were used as the basis of the co-registration. Figure 1 shows the workflow for the co-registration steps. For matching p_A_O_2_ and ADC results, the Advanced Normalization Tool (ANTs) 3D registration software [37] was used. The final p_A_O_2_ slice scans were down-sampled from a 16.5 mm slice thickness to a 40 mm slice thickness and cropped following the procedure of Hughes et al. [38]. The resulting images were converted to 5 coronal slices matching the anatomy covered in the ADC scans

### 2.5. Regional Divisions Using Proton MRI and ^3^He Ventilation Data

**Figure 2:**
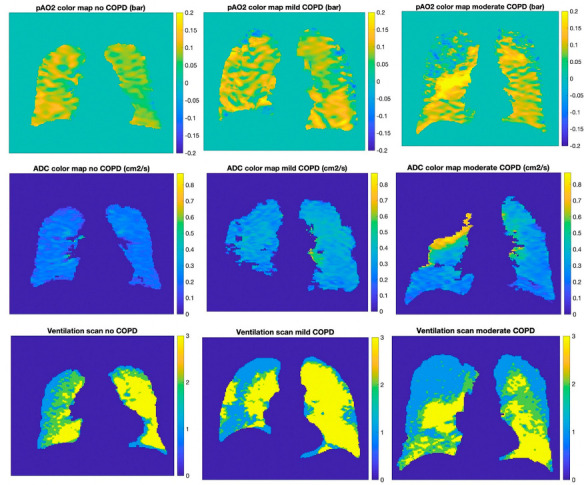
Color maps of three subjects with different levels of disease severity. Left column: subject with no COPD, center column: mild COPD, right column: severe COPD; from top to bottom: p_A_O_2_ color map, ADC color map, ventilation scan. On the ventilation scans blue corresponds to non-ventilated regions, green hypo-ventilated and yellow normal.

Ventilation masks divided the lung into three separate ventilation regions, namely normal ventilation, hypo (or low) ventilation, and no ventilation regions. Non-ventilated regions were defined as being within 2.6 SD of the mean heart signal (S_heart_), covering 99.5% of the Z score data. Similarly, ventilated regions were set to be within 2.6 SD of the heart signal plus one-quarter of the difference between the trachea and heart signal, namely S_ventilated_ = S_heart_ + 0.25 (S_trachea_ − S_heart_). Any remaining lung regions were labeled hypo-ventilated. Figure 2 presents an example of a lung slice with three ventilation regions differentiated by three different mask colors. These same masks were first produced and used in a study of low ventilation defect percentages associated with cardiac function [39].

A separate analysis in which the coronal slices were divided into six zones defined by cranial-caudal thirds (left versus right as well as upper, middle and lower) allowed for a comparison of the regional p_A_O_2_ results for different pulmonary emphysema subtypes evaluated from a visual CT analysis [4]. Coronal proton MRI images were used to find the divisions between the upper, middle and lower zones. The upper zone was chosen as extending from the apex to the mid-aortic arch. The lower zone extended from the mid-entry level of the most inferior pulmonary vein to the diaphragm. The middle zone incorporated the remaining region between the upper and lower zones.

### 2.6. Non-Physiologic Results for p_A_O_2_

A significant challenge for studying p_A_O_2_ in moderate/severe COPD participants is the ^3^He gas mixing and voxel to voxel transport between acquisitions, resulting in negative p_A_O_2_ values. Non-physiologic values are defined as values resulting from gas mixing during the measurement time and not an actual partial pressure measurement, namely p_A_O_2_ values smaller than 0.

### 2.7. CT Measures

All participants underwent CT imaging following the MESA Lung protocol at a fixed mAs [40]. Percent emphysema is defined as the percentage of voxels in the lung field with Hounsfield units below −950 [40].

Prior work in the MESA COPD Study qualitatively assessed the presence and severity of traditional emphysema subtypes [4], namely centrilobular (CLE), panlobular (PLE) and paraseptal emphysema (PSE), as identified by radiologists using CT scans. The lung was divided into six regions, and a severity score (0–100) was assigned for each region. A global pulmonary emphysema subtypes severity score was calculated for each participant by summing the severity scores of each of the six regions.

### 2.8. Pulmonary Function Testing

Spirometry was performed following the MESA Lung protocol and contemporary American Thoracic Society/European Respiratory Society standards [40]. In addition, DLCO measures collected at the baseline MESA COPD visit in November 2009 [4].

### 2.9. Statistical Analysis

Dichotomous variables are presented as proportions and continuous variables are presented as means with standard deviations (SD) unless otherwise indicated. Mean, SD, and percent negative p_A_O_2_ values were calculated across six lung regions for each coronal slice and then combined into volume-weighted global p_A_O_2_ measures per subject. Non-physiologic p_A_O_2_ measures were included in mean and SD calculations and also used as a separate metric, %negative, to show the percentage of measurements that were below zero to further describe impairment in blood oxygenation.

Spearman’s correlation coefficients, with Fisher’s Z transformation, were used to assess correlations between p_A_O_2_ measures and percent emphysema and ADC mean. The Mann-Whitney U test was used to assess differences in percentage negative p_A_O_2_ for different ventilation states across COPD status. Inverse probability weights were used to make results comparable to the general population, based on the known likelihood of selection into the study sample.

Generalized linear models adjusting for age, sex, race/ethnicity, and smoking status were used to assess associations between global p_A_O_2_ values and percent emphysema, visual emphysema severity, and pulmonary emphysema subtypes. We refer to models that adjust for these covariates as “Model 1”. Models were additionally adjusted for percent predicted FEV_1_ (“Model 2”). Regional associations were assessed using linear mixed models with random intercepts by lung region. In order to obtain estimates of emphysema subtype prevalence in the source population, analyses were weighted by the ratio of COPD prevalence in the source study to that in the MESA COPD Study, as previously described [41,42]. A two-tailed *p* value < 0.05 was considered statistically significant. All analyses were performed using SAS 9.4 (SAS Institute, Cary, NC, USA) and R (version 4.0.4).

## 3. Results

Of 200 participants who participated in the MESA COPD study and were eligible for hyperpolarized gas imaging, 56 consented and underwent hyperpolarized gas imaging. Of these, p_A_O_2_ was able to be measured for 54 participants and ADC for 50 (Supplementary Figure S1).

### 3.1. Global p_A_O_2_ versus ADC

Figure 2 shows p_A_O_2_ and ADC color maps of a central coronal section for three participants: one without COPD, one with mild COPD, and one with moderate COPD. The map for the subject without COPD presents a homogeneous distribution (ADC ~0.3 cm^2^/s), whereas the frequency of high ADC regions generally is higher for participants with increasing severity of COPD. Regions of high ADC have been shown to correlate with regions of emphysema, which occurs together with COPD [5]. The mean p_A_O_2_ results for the three participants over the full lung are comparable; however, the p_A_O_2_ heterogeneity in the more severe COPD subject is visibly higher. Although there is a substantial scatter in the results across all participants, these general trends persist throughout the patient population and have been observed in previous studies [35]. When compared to the level of ventilation, regions of negative p_A_O_2_ tend to overlap with regions of no ventilation. Further, as COPD severity increases, regions of normal ventilation become smaller as those with hypo- and non-ventilation increase.

**Figure 3:**
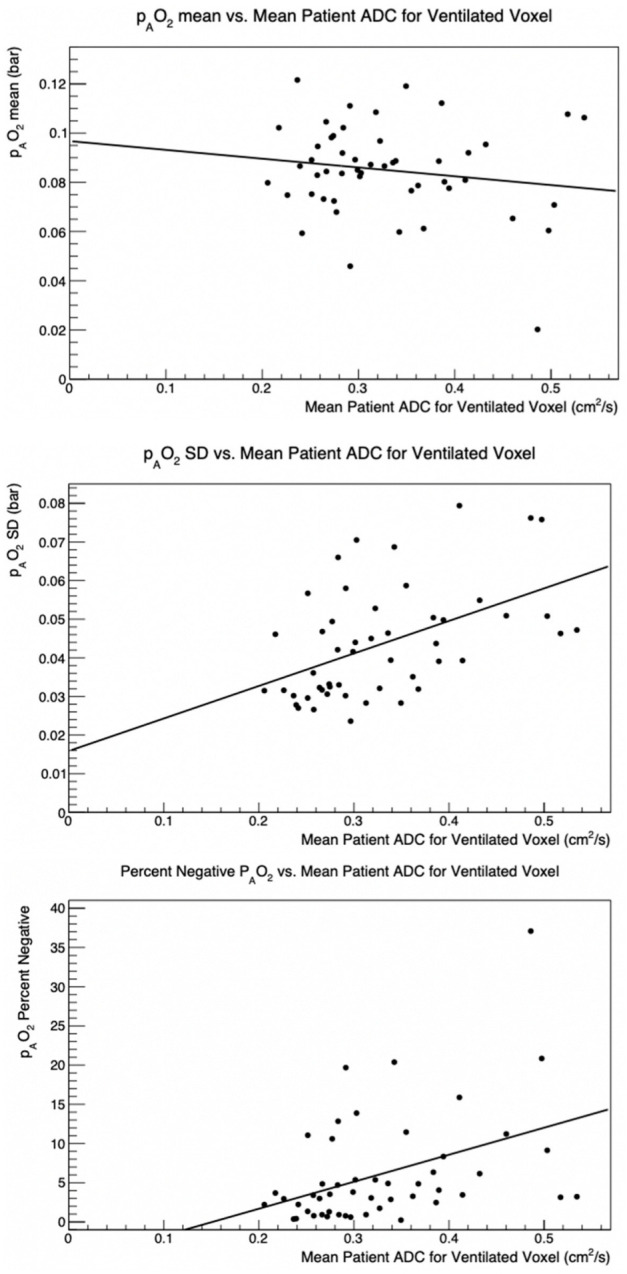
p_A_O_2_ mean vs. ADC mean (top), p_A_O_2_ SD vs. ADC mean (center) Percent negative p_A_O_2_ vs. ADC mean (bottom). Each measurement corresponds to an individual participant imaged with polarized ^3^He from the MESA COPD Study.

Figure 3 shows a compilation of the mean and SD p_A_O_2_ as a function of the mean ADC value for 50 participants imaged with HP ^3^He. The data in Figure 3 were matched to the p_A_O_2_ results. Although there is a significant spread in results, there is a distinct trend of increasing p_A_O_2_ SD for participants with higher ADC values, whereas the mean p_A_O_2_ values appears to be largely uncorrelated to the mean ADC value. A doubling in the ADC values results in approximately a doubling of the p_A_O_2_ SD (Figure 3, center).

The overall mean ADC value was 0.302 ± 0.061 cm^2^/s. The mean p_A_O_2_ value was 0.091 ± 0.034 bar. Global p_A_O_2_ SD was strongly correlated with ADC mean (Table 3). When negative values were excluded from p_A_O_2_ mean and SD calculations, though numerical results changed slightly, our conclusions remained the same.

### 3.2. Negative p_A_O_2_ Results

Figure 3 presents the negative p_A_O_2_ percentage as a function of mean ADC per subject for the study. The large increase in negative p_A_O_2_ with increased ADC is evident. Similar to the p_A_O_2_ SD, there is a strong association between the negative p_A_O_2_ percentage and the mean ADC, summarized in Table 3. Figure 2 shows the overlap of the negative regions with the non-ventilated regions: regions with negative p_A_O_2_ tend to overlap with regions where no ADC was measured i.e., there was no ventilation.

### 3.3. Global p_A_O_2_ and Percent Emphysema 

Table 3 shows strong correlations between percent emphysema and p_A_O_2_ SD and percent negative p_A_O_2_. Metrics showing an increase in heterogeneity of p_A_O_2_ values, shown by the SD and the percent negative, is strongly correlated with both %emphysema and ADC.

### 3.4. Measurements of p_A_O_2_ in Different Ventilation Regions

Mean values for the p_A_O_2_ measurements per subject for the three different ventilation regions have been compiled. The overall mean p_A_O_2_ per subject is 0.105 ± 0.026 (SD) within the normal ventilation regions, 0.096 ± 0.025 (SD) within the hypo-ventilation regions, and 0.094 ± 0.019 (SD) within the non-ventilated regions. The observed difference in p_A_O_2_ with ventilation state is statistically significant.

**Figure 4:**
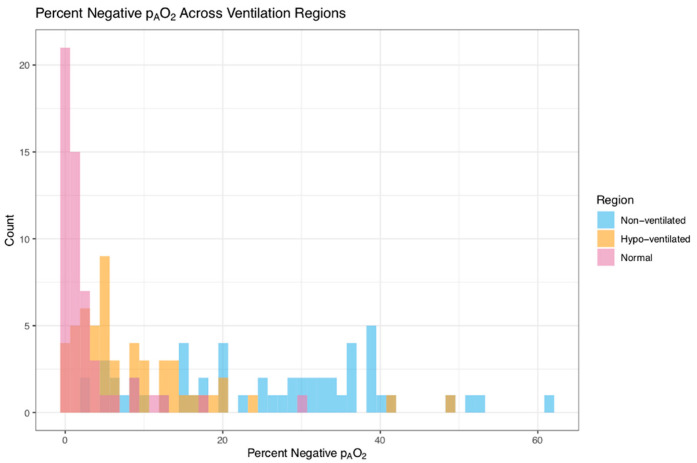
Average percent negative p_A_O_2_ per subject in normal, hypo-, and non-ventilated for a single subject.

Figure 4 provides the percentage of negative p_A_O_2_ values for the three different ventilation types. There is a marked difference between the different ventilation regions with the range of values extending to high percentages for the non-ventilated region. The median [interquartile range, IQR] for percent negative p_A_O_2_ is 1.3% [0.3, 2.5] for the normal ventilated region, 5.1% [3.0, 12.2] for the hypo-ventilated region and 27.0% [15.2, 36.4] for the non-ventilated region. 

### 3.5. Global p_A_O_2_ Measurements Compared to Carbon Monoxide Diffusion Capacity

The p_A_O_2_ SD was significantly associated with DLCO in both unadjusted and adjusted models (*p* = 0.007 and *p* = 0.04 respectively), supporting the utility of p_A_O_2_ SD as a potential marker of diffusion impairment [35] (Table 4). DLCO measurements were collected at the baseline MESA COPD exam among 34 of the 54 participants. Negative p_A_O_2_ was only found to be significantly associated with DLCO in an unadjusted model (*p* = 0.01).

### 3.6. Regional p_A_O_2_ Measurements Compared to Traditional Emphysema Subtypes

The median [interquartile range, IQR] of the regional mean p_A_O_2_ measures across the six regions of the lung are as follows: upper left, 0.076 [0.064, 0.089]; upper right, 0.086 [0.069, 0.101]; middle left, 0.083 [0.073, 0.095]; middle right, 0.100 [0.091, 0.111]; lower left, 0.087 [0.077, 0.105]; and lower right, 0.107 [0.097, 0.122]. Detailed results including information on the emphysema severity per region is presented in the Supplementary Table S1. One observes a clear increase in p_A_O_2_ mean from apical to basal and a consistently higher p_A_O_2_ mean in the right lung compared to the left lung for the studied population.

Table 5 provides the associations between the regional p_A_O_2_ measures compared to percent emphysema and pulmonary emphysema subtypes presence or severity score. All models reveal an association between p_A_O_2_ SD and the pulmonary emphysema subtypes severity score, while the percent negative p_A_O_2_ shows an association with the pulmonary emphysema subtypes severity score for the unadjusted model and Model 1, but not Model 2. The p_A_O_2_ SD appears to be mildly sensitive to the presence of PSE both for the unadjusted model as well as Model 1. The association is not significant when additionally adjusted for percent predicted FEV_1_ in Model 2. 

## 4. Discussion

HP ^3^He MRI measurements of lung p_A_O_2_ reveal a marked increase in heterogeneity of oxygenation, as assessed by p_A_O_2_ SD, with increasing % emphysema (measured on CT [41]). The strong correlation between ADC and percent emphysema confirms that ADC is an appropriate metric for determining the severity of damage caused by the disease. Similarly, the presence of non-physiologic, negative p_A_O_2_ measurements is strongly associated with increased emphysema, as well as in comparison to HP ^3^He ADC measurements. The overlap of non-ventilated regions and regions with high percentages of negative values is significant.

p_A_O_2_ SD and negative p_A_O_2_ are also strongly associated with pulmonary emphysema subtype severity scores [4]. An investigatory look at p_A_O_2_ SD and negative p_A_O_2_ also reveal mild sensitivity to the severity of PSE Since PSE is present in only in 9% of the MESA COPD population [4], this presents p_A_O_2_ SD as a potentially beneficial and safe biomarker for PSE detection and possibly other emphysema subtypes. Larger sample sizes would be worthwhile for future studies.

The p_A_O_2_ SD results are consistent with an earlier study [35] in 7 COPD patients. The current results also reveal no sensitivity to the p_A_O_2_ mean in either a global or regional lung study. Other studies have also found large negative p_A_O_2_ regions for patients with severe COPD [18], but did not quantify the effect in mild to moderate COPD, nor compare to emphysema measurements from CT on the same subjects.

Since p_A_O_2_ depends upon the balance between oxygen replenishment from inhalation and oxygen diffusion into blood, it is expected that degradation in oxygen diffusion from emphysema would change p_A_O_2_. Therefore, DLCO which reflects the absorption of carbon monoxide could correlate with p_A_O_2_ [35]. We do observe correlations between p_A_O_2_ and DLCO measurements as shown in Table 4. In the MESA COPD study, DLCO measurements were not collected at the same time as the HP ^3^He measurements. However, DLCO measurements were performed approximately five years earlier on 34 overlapping subjects in MESA COPD I [31]. In spite of the different time measurements and reduced number of subjects, a noteworthy association exists between global p_A_O_2_ SD and decreased DLCO and the mean p_A_O_2_ was decreased in subjects with impaired DLCO as expected.

The primary motivation for HP ^3^He MRI studies of p_A_O_2_ in COPD is to determine the actual p_A_O_2_ mapping. HP ^3^He MRI measurements of regional p_A_O_2_ in lung parenchyma face several technical hurdles: gas mixing between neighboring voxels produces non-physiological results; RF depolarization effects; body motion during the 20 s breath-hold disturbs voxel registration from one measurement to the next so reproducibility is a continual challenge. For high field MRI, in this case 3T, imaging artifacts appear, and corrections may need to be implemented for the lost data. The correction for the RF depolarization from the scanner has been an issue since the first p_A_O_2_ measurements [17]. The significant fluctuations in the voxel to voxel only allow for bounding the absolute p_A_O_2_ determination from RF depolarization to be a ~ ±10% systematic uncertainty.

Non-physiological values are a challenge to the interpretation of p_A_O_2_ measurements from HP noble gas MRI. These appear in three forms. As has been discussed previously [18,19], mixing from different regions can result in negative p_A_O_2_ values. In addition, mixing can also create the opposite effect, namely that p_A_O_2_ results with values greater than the oxygen partial pressure of air can be recorded. A negligible number of voxels with a value greater than 0.2 bar were recorded and as such were not considered further during the analyses. Negative values reflect gas moving into that voxel during scanning, which could be indicative of delayed ventilation [18]. The effect of negative p_A_O_2_ values on neighboring positive regions needs to be considered as well since p_A_O_2_ signal is leaving the voxel of interest, making the signal decay greater than it would be without the gas movement, leading to falsely elevated p_A_O_2_ values. Finally, very low values of p_A_O_2_, namely regions of hypo-ventilation, were found and might be influenced by the same issues that cause negative p_A_O_2_. Care needs to be taken with the interpretation of hypo-ventilation regions, especially in the vicinity of negative p_A_O_2_ regions. As a systematic check, the analysis was also carried out in which only non-negative values were used in the p_A_O_2_ calculations, and the substantive conclusions remained the same. Unlike the presence of delayed ventilation in patients with severe COPD, these negative p_A_O_2_ regions had not been observed in patients with mild COPD in previous studies of p_A_O_2_, an indication that HP MRI provides insight into earlier stages of the disease.

Future modeling and simulations of intervoxel gas mixing in patients with moderate or severe COPD could be enormously valuable additions to gain back the measurement loss of true p_A_O_2_. A potential beneficial future research direction is to compare regionally negative p_A_O_2_ regions to the existence of blebs, identified by CT.

## 5. Conclusions

First measurements of p_A_O_2_ using polarized ^3^He on 54 participants from the MESA COPD study has been performed and compared to pulmonary functional tests and CT studies. To our knowledge, this represents the largest study of COPD subjects to determine p_A_O_2_ values using HP ^3^He MRI_._ Unlike previous studies which were conducted on small sample sizes with extreme cases, this study was a population-based nested case-control study looking at a wide range of disease severity—from none to mild to severe—as is reflected in the real world. We find a significant and quantifiable increase in the p_A_O_2_ heterogeneity as a function of lung damage, compared to ^3^He MRI ADC measurements as well as compared to percent lung emphysematous damage determined in CT studies [41]. A first look is taken comparing p_A_O_2_ measures to pulmonary emphysema subtypes. The large percentage of non-physiological p_A_O_2_ results in some subjects, which primarily originate from non-ventilated regions, represents both a challenge to determining the true p_A_O_2_ as well as a potential biomarker for the disease progression.

Present and future research using polarized noble gas MRI are aimed primarily at ramping up with polarized ^129^Xe. HP ^129^Xe MRI has a number of important advantages compared to ^3^He, including being significantly cheaper and more readily available. Initial studies on p_A_O_2_ measurements using ^129^Xe with COPD participants have been performed [43]. Nevertheless, the intrinsically lower SNR for ^129^Xe will remain a challenge to the method. 

Future efforts to model the systematic effects in p_A_O_2_ measurements for HP ^3^He as a function of regional lung damage could be enormously valuable and a viable future research direction. Determining regional ventilation/perfusion in COPD patients using polarized noble gas MRI [11] is still an achievable goal, despite the increased challenge arising from damage caused by disease. 

## Figures and Tables

**Table 1:** Characteristics of the study population.

Characteristic	All Participants (*n* = 54)
Age, years—mean ± SD	73.3 ± 7.4
Female—N (%)	22 (40.7%)
Race/Ethnicity—N (%)	
White	28 (51.9%)
Black	17 (31.5%)
Hispanic	9 (16.6%)
Height, cm—mean ± SD	175.8 ± 4.8
Weight, lb—mean ± SD	75.8 ± 14.2
Smoking status—N (%)	
Former smoker	38 (70.4%)
Current smoker	16 (29.6%)
Pack-years—mean ± SD	41.3 ± 23.0
Educational attainment—N (%)	
<High school degree	15 (27.8%)
College degree	24 (44.4%)
Some college/2-year degree	15 (27.8%)
*Spirometry (post-bronchodilator)*	
FEV_1_ (mL)—mean ± SD, *n* = 53	2157 ± 577
FVC (mL)—mean ± SD, *n* = 53	3326 ± 965
FEV_1_/FVC—mean ± SD, *n* = 53	0.67 ± 0.13
COPD—No. (%)	29 (53.7%)
COPD severity—N (%)	
None	25 (46.3%)
Mild	13 (24.1%)
Moderate	16 (29.6%)
*CT measures*	
Total lung volume (mL)—mean ± SD	5460 ± 1353
Total tissue volume (mL)—mean ± SD	835 ± 176
Total air volume (mL)—mean ± SD	4625 ± 1220
% emphysema −950 HU—median [IQR]	2.1 [0.9, 7.1]
Visual emphysema severity (%) ^1^—median [IQR]	0.24 [0, 2.31]
*DLCO measures, n* = *34*	
DLCO (%)—mean ± SD	18.1 ± 4.9
*^3^He MRI measures*	
p_A_O_2_ mean (bar)—median [IQR]	0.093 [0.084, 0.103]
p_A_O_2_ SD (bar)—median [IQR]	0.052 [0.037, 0.064]
p_A_O_2_ %negative—median [IQR]	4.9 [2.1, 7.5]
ADC mean (cm^2^/s)—median [IQR]	0.302 [0.267, 0.383]

^1^ Calculated as the sum of the severity scores for centrilobular, panlobular, and paraseptal emphysema; DLCO, diffusing capacity of carbon monoxide. COPD status was defined as: post-bronchodilator FEV_1_/FVC < 0.7. COPD severity was defined as: Mild: %-predicted FEV_1_ ≥ 80; Moderate: 50 ≤ %-predicted FEV_1_ < 80.

**Table 2:** Gas composition for imaging sequences.

Scan	^3^He	N_2_	Breath-Hold
Calibration	150 cc	-	-
Ventilation	300 cc	700 cc	TLC
ADC	500 cc	500 cc	TLC
p_A_O_2_	350 cc	650 cc	FRC + 1 L

**Table 3:** Spearman’s correlation coefficients between emphysema measures and p_A_O_2_ measures.

		Global p_A_O_2_ Mean ^1^		Global p_A_O_2_ SD ^1^		Global p_A_O_2_ %Negative ^1^		ADC Mean ^1^	
Measure	N	*ρ* (95% CI)	*p*	*ρ* (95% CI)	*p*	*ρ* (95% CI)	*p*	*ρ* (95% CI)	*p*
%emphysema −950 HU	54	−0.08 (−0.34, 0.20)	0.59	0.48 (0.24, 0.66)	0.0002 *	0.50 (0.27, 0.68)	<0.0001 *	0.81 (0.68, 0.89)	<0.0001 *
Visual emphysema severity (%) ^2^	50	0.24 (−0.05, 0.48)	0.10	0.38 (0.11, 0.59)	0.006 *	0.32 (0.04, 0.55)	0.02 *	0.66 (0.44, 0.79)	<0.0001 *
ADC mean (cm^2^/s)	50	−0.07 (−0.34, 0.21)	0.61	0.53 (0.29, 0.70)	<0.0001 *	0.45 (0.20, 0.65)	0.0008 *	-	-

^1^ Per 0.01 change. ^2^ Calculated as the sum of the severity scores for centrilobular, panlobular, and paraseptal emphysema. * *p*-value < 0.05. ADC, apparent diffusion coefficient; HU, Hounsfield units; p_A_O_2_, partial pressure of oxygen; *ρ*, Spearman’s correlation coefficient; SD, standard deviation, Fisher’s z-transformation was used to calculate 95% confidence intervals.

**Table 4:** Global associations with p_A_O_2_ measures.

		p_A_O_2_ Mean		p_A_O_2_ SD		p_A_O_2_ %Negative	
Exposure	N	*β* (95% CI)	*p*	*β* (95% CI)	*p*	*β* (95% CI)	*p*
%emphysema −950 HU							
Unadjusted	54	−0.0007 (−0.002, 0.0005)	0.26	0.002 (0.0009, 0.003)	0.0002 *	0.57 (0.29, 0.86)	0.0002 *
Model 1	54	−0.0003 (−0.002, 0.0009)	0.62	0.001 (0.0003, 0.002)	0.005 *	0.37 (0.11, 0.63)	0.007 *
Model 2	53	−0.0001 (−0.001, 0.001)	0.85	0.001 (0.0003, 0.002)	0.006 *	0.32 (0.05, 0.60)	0.02 *
Visual emphysema severity (%) ^1^							
Unadjusted	50	−0.0002 (−0.001, 0.0006)	0.64	0.0008 (0.0002, 0.001)	0.01 *	0.29 (0.10, 0.48)	0.004 *
Model 1	50	−0.00005 (−0.0008, 0.0007)	0.90	0.0005 (0.0001, 0.001)	0.02 *	0.22 (0.05, 0.38)	0.01 *
Model 2	49	0.00006 (−0.0008, 0.0009)	0.89	0.0005 (0.00003, 0.001)	0.04 *	0.19 (0.01, 0.36)	0.04 *
ADC ^2^							
Unadjusted	50	−0.0005 (−0.001, 0.0002)	0.17	0.0008 (0.0003, 0.001)	0.003 *	0.36 (0.12, 0.61)	0.004 *
Model 1	50	−0.0004 (−0.001, 0.0004)	0.33	0.0007 (0.0002, 0.001)	0.005 *	0.30 (0.08, 0.53)	0.01 *
Model 2	49	−0.0002 (−0.001, 0.0006)	0.63	0.0006 (0.0002, 0.001)	0.01 *	0.24 (0.02, 0.47)	0.04 *
DLCO							
Unadjusted	34	−0.0003 (−0.002, 0.001)	0.65	0.001 (0.0003, 0.002)	0.007 *	0.23 (0.05, 0.41)	0.01 *
Model 1	34	−0.0001 (−0.002, 0.002)	0.93	−0.001 (−0.002, −0.00003)	0.04 *	−0.22 (−0.46, 0.01)	0.06
Model 2	34	−0.0001 (−0.002, 0.002)	0.91	−0.001 (−0.002, −0.00004)	0.04 *	−0.22 (−0.45, 0.01)	0.06

^1^ Calculated as the sum of the severity scores for centrilobular, panlobular, and paraseptal emphysema. ^2^ Per 0.01 change. * *p*-value < 0.05. ADC, apparent diffusion coefficient; HU, Hounsfield units; p_A_O_2_, partial pressure of oxygen; SD, standard deviation. Unadjusted: (weighted). Model 1: adjusted for age, sex, race/ethnicity, cigarette smoking status (weighted). Model 2: Model 1 + %-predicted FEV1 (weighted). Generalized linear models were used, and participants were weighted on the inverse ratio of probability of selection.

**Table 5:** Regional associations between percent emphysema or pulmonary emphysema subtype severity and p_A_O_2_ measures.

	p_A_O_2_ Mean		p_A_O_2_ SD		p_A_O_2_ %Negative	
Exposure	*β* (95% CI)	*p*	*β* (95% CI)	*p*	*β* (95% CI)	*p*
%emphysema, log-transformed ^1^						
Unadjusted (*n* = 54)	0.0001 (−0.003, 0.003)	0.94	0.0006 (−0.002, 0.003)	0.61	0.43 (−0.44, 1.30)	0.33
Model 1 (*n* = 54)	0.0008 (−0.002, 0.004)	0.59	0.00002 (−0.002, 0.002)	0.99	0.15 (−0.71, 1.01)	0.73
Model 2 (*n* = 53)	0.001 (−0.002, 0.004)	0.39	−0.0004 (−0.003, 0.002)	0.74	−0.05 (−0.92, 0.81)	0.90
Visual emphysema severity (%), log-transformed ^1,2^						
Unadjusted (*n* = 50)	−0.0007 (−0.005, 0.004)	0.73	0.006 (0.002, 0.009)	0.001 *	1.61 (0.36, 2.86)	0.01 *
Model 1 (*n* = 50)	−0.0005 (−0.005, 0.004)	0.80	0.005 (0.002, 0.008)	0.003 *	1.46 (0.29, 2.63)	0.01 *
Model 2 (*n* = 49)	0.0009 (−0.004, 0.005)	0.69	0.003 (0.0001, 0.007)	0.04 *	0.87 (−0.38, 2.11)	0.17
Severity scores of pulmonary emphysema subtypes, log-transformed ^1,3^						
Unadjusted (*n* = 50)						
CLE severity	0.003 (−0.004, 0.009)	0.38	−0.0006 (−0.006, 0.005)	0.82	−0.42 (−2.31, 1.48)	0.67
PLE severity	0.001 (−0.005, 0.008)	0.68	0.002 (−0.002, 0.007)	0.33	0.96 (−0.84, 2.76)	0.30
PSE severity	−0.006 (−0.01, 0.002)	0.13	0.008 (0.002, 0.01)	0.005 *	2.29 (0.22, 4.37)	0.03 *
Model 1 (*n* = 50)						
CLE severity	0.003 (−0.004, 0.009)	0.39	−0.0001 (−0.005, 0.005)	0.97	−0.20 (−2.02, 1.62)	0.83
PLE severity	0.001 (−0.005, 0.007)	0.76	0.002 (−0.002, 0.007)	0.29	1.31 (−0.46, 3.08)	0.15
PSE severity	−0.004 (−0.01, 0.003)	0.24	0.006 (0.0002, 0.01)	0.04 *	1.53 (−0.54, 3.60)	0.15
Model 2 (*n* = 49)						
CLE severity	0.004 (−0.003, 0.01)	0.29	−0.001 (−0.006, 0.004)	0.69	−0.56 (−2.42, 1.30)	0.56
PLE severity	0.0009 (−0.005, 0.007)	0.77	0.003 (−0.002, 0.007)	0.28	1.33 (−0.44, 3.09)	0.14
PSE severity	−0.003 (−0.01, 0.005)	0.50	0.004 (−0.002, 0.01)	0.17	0.69 (−1.45, 2.83)	0.53

^1^ Continuous exposure variables (i.e., %emphysema, pulmonary emphysema subtype severity scores) were log-transformed to achieve approximately normal distributions of residuals. ^2^ Calculated as the sum of the severity scores for centrilobular, panlobular, and paraseptal emphysema. ^3^ Pulmonary emphysema subtypes are adjusted for each other. * *p*-value < 0.05. CLE, centrilobular emphysema; PLE, panlobular emphysema; PSE, paraseptal emphysema. Mixed models with a random intercept, variance component (VC) structure, and Kenward-Roger’s. approximation of the degrees of freedom were used. Participants were weighted on the inverse ratio of probability of selection. Pulmonary emphysema subtypes were qualitatively assessed at the baseline MESA COPD exam and scored from 0–100. Two participants were excluded due to non-physiological results. Unadjusted: (weighted). Model 1: adjusted for age, sex, race/ethnicity, and cigarette smoking status (weighted). Model 2: Model 1 + %-predicted FEV1 (weighted).

## Data Availability

Not applicable.

## Supplementary Materials

The following supporting information can be downloaded at: https://www.mdpi.com/article/10.3390/tomography8050190/s1, Figure S1: MESA ^3^He MRI Participant Flow Chart. Table S1: Distributions of regional measures (n = 54).
