# Supplementary material for: Mapping Alveolar Oxygen Partial Pressure in COPD Using Hyperpolarized Helium-3: The Multi-Ethnic Study of Atherosclerosis (MESA) COPD Study
Source: Tomography. 2022 Sep 13;8(5):2268–84. doi: 10.3390/tomography8050190 (PMC9498778; doi:10.3390/tomography8050190)
Supplement: Supplementary file 1 [file tomography-08-00190-s001.zip › tomography-1873651-supplementary.pdf]

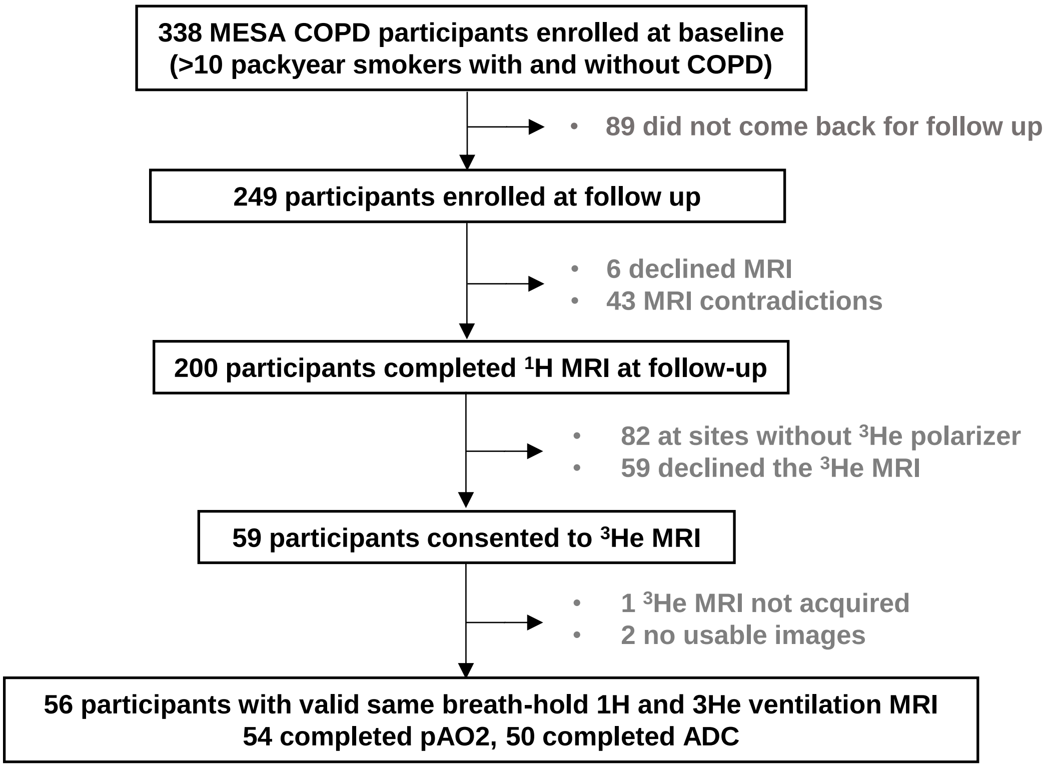

Supplementary Figure S1 MESA ^{3}He MRI Participant Flow Chart.

Characteristic	Upper left	Upper right	Middle left	Middle right	Lower left	Lower right
p_AO_2 mean, all values[1] – median [IQR]	0.076 [0.064, 0.089]	0.086 [0.069, 0.101]	0.083 [0.073, 0.095]	0.100 [0.091, 0.111]	0.087 [0.077, 0.105]	0.107 [0.097, 0.122]
p_AO_2 SD, all values[1] – median [IQR]	0.057 [0.037, 0.073]	0.057 [0.042, 0.076]	0.044 [0.031, 0.065]	0.048 [0.036, 0.064]	0.053 [0.036, 0.086]	0.048 [0.035, 0.081]
p_AO_2 %negative – median [IQR]	7.5 [3.4, 15.0]	5.5 [2.5, 13.4]	3.8 [1.6, 7.5]	2.9 [1.1, 5.5]	3.9 [1.2, 10.8]	2.6 [0.9, 7.9]
%emphysema -950 HU – median [IQR]	2.20 [0.47, 5.29]	1.90 [0.39, 4.35]	2.03 [0.89, 5.30]	1.85 [0.87, 5.82]	1.72 [0.97, 6.39]	1.90 [0.41, 4.95]
Visual emphysema severity (%)[2] – median [IQR]	0.06 [0, 3.20]	0.56 [0, 3.83]	0.15 [0, 1.40]	0.21 [0, 3.67]	0 [0, 1.67]	0 [0, 0.50]
Centrilobular emphysema, n=50						
Severity (0-100) – median [IQR], range	0 [0, 1.22] Range: 0-59.00	0.23 [0, 2.63] Range: 0-60.00	0 [0, 0.42] Range: 0-22.50	0 [0, 1.75] Range: 0-25.00	0 [0, 0.33] Range: 0-6.00	0 [0, 0.23] Range: 0-11.00
Panlobular emphysema, n=50						
Severity (0-100) – median [IQR], range	0 [0, 0] Range: 0-34.60	0 [0, 0] Range: 0-44.80	0 [0, 0] Range: 0-11.90	0 [0, 0] Range: 0-10.00	0 [0, 0] Range: 0-20.00	0 [0, 0] Range: 0-25.00
Paraseptal emphysema, n=50						

Severity (0-100) – median [IQR], range	0 [0, 0.50] Range: 0-56.67	0 [0, 1.00] Range: 0-98.33	0 [0, 1.13] Range: 0-20.33	0 [0, 0.83] Range: 0-51.67	0 [0, 0] Range: 0-6.00	0 [0, 0] Range: 0-18.33

[1]Per 0.01 change

[2]Calculated as the sum of the severity scores for centrilobular, panlobular, and paraseptal emphysema

IQR, interquartile range.

Supplementary Table S1: Distributions of regional measures (n=54)
